# Supplementary material for: Supramolecular Nature of Multicomponent Crystals Formed from 2,2′-Thiodiacetic Acid with 2,6-Diaminopurine or N9-(2-Hydroxyethyl)adenine
Source: Int J Mol Sci. 2023 Dec 12;24(24):17381. doi: 10.3390/ijms242417381 (PMC10743529; doi:10.3390/ijms242417381)

International Journal of Molecular Sciences

Supplementary material

Supramolecular nature of multicomponent crystals formed from 2,2'-thiodiacetic acid with 2,6-diaminopurine or N9-(2-Hydroxyethyl)adenine

Jeannette Carolina Belmont-Sánchez,[1] Duane Choquesillo-Lazarte,[2] María Eugenia García-Rubiño,[3] Antonio Matilla-Hernández,[1] Juan Niclós-Gutiérrez,[1] Alfonso Castiñeiras,[4,*] Antonio Frontera[5]

Contents

S1. Crystals of [(H9Heade$^+$)(Htda$^-$)], **1**. 2
S2. FT-IR spectrum of [(H9Heade$^+$)(Htda$^-$)], **1**. 3
S3. FT-IR spectrum of [$(H_2dap^+)_2(tda^{2-})$]·$2H_2O$, **2**. 3
S4. Thermogravimetric analysis [(H9Heade$^+$)(Htda$^-$)], **1**. 4
S5. Thermogravimetric analysis [$(H_2dap^+)_2(tda^{2-})$]·$2H_2O$, **2**. 10
S6. Thermogravimetric analysis of the reagents used in this work. 16
S6.1. TGA of 2,2'-Thiodiacetic acid, H_2tda (powder, 98 %). 16
S6.2. TGA of 2,6-diaminopurine, Hdap (powder, 98 %). 17
S6.3. TGA of N9-(2-hydroxyethyl)adenine, 9heade (powder, 98%). 18
S7. DSC curve for [(H9Heade$^+$)(Htda$^-$)], **1**. 19
S8. DSC curve for [$(H_2dap^+)_2(tda^{2-})$]·$2H_2O$, **2**. 20
S9. 1H NMR spectrum in D_2O of **1.** **21**
S10. 1H NMR spectrum in D_2O of **2.** **21**
S11. 13C NMR spectrum in D_2O of **1.** **22**
S12. 13C NMR spectrum in D_2O of **2.** **22**

SM1. Crystals of [(H9Heade^{+})(Htda^{-})], **1**.

Three photos taken from dissolution using polarized light.

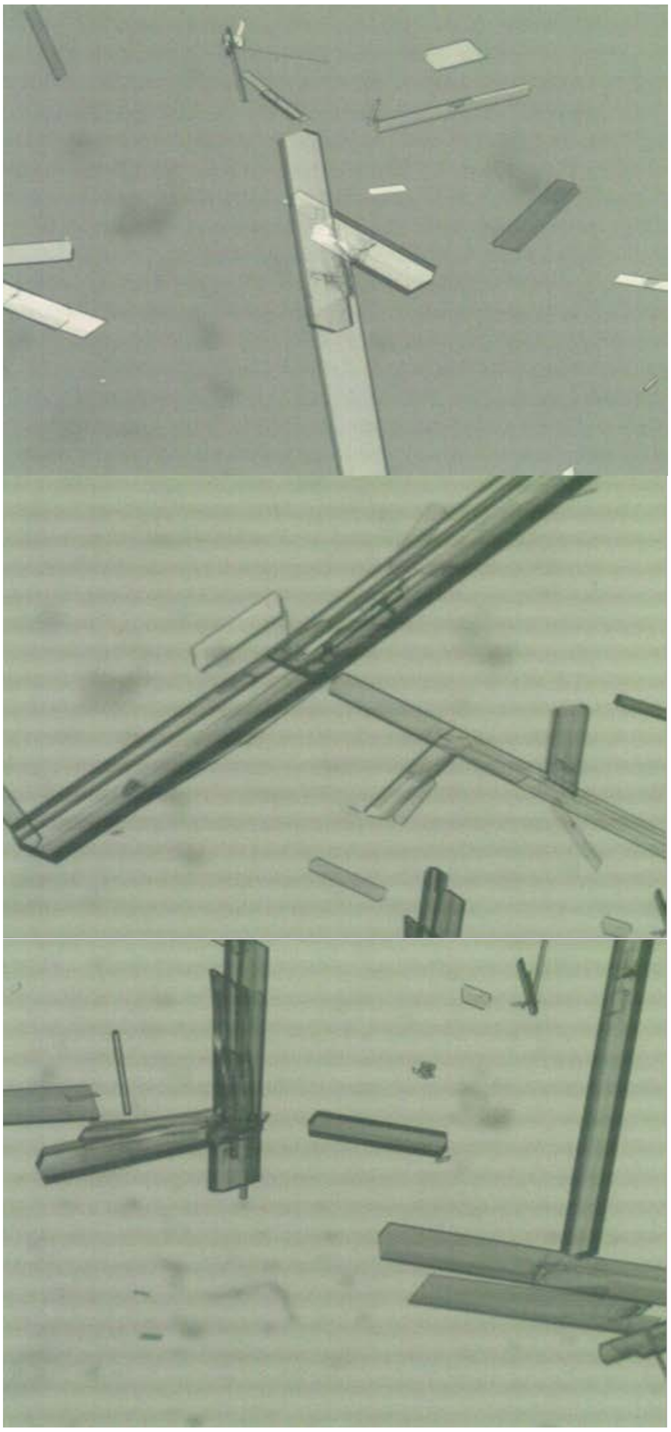

S2. FT-IR spectrum of [(H9Heade^{+})(Htda^{-})], **1**.

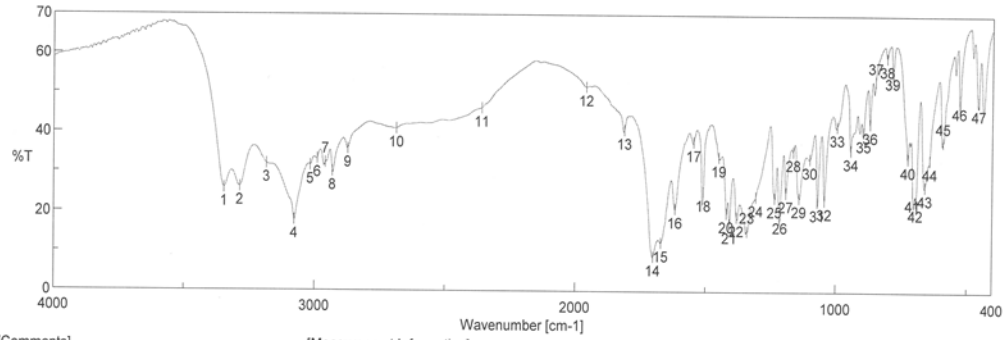

[Comments]		[Measurement Information]	
Sample name		Model Name	FT/IR-6300typeA
Comment		Serial Number	A014961024
User			
Division		Measurement Date	24/09/2020 13:03
Company	Universidad de Granada		
		Light Source	Standard
[Detailed Information]		Detector	TGS
Creation date	24/09/2020 13:04	Accumulation	250
Date modified	24/09/2020 13:15	Resolution	4 cm-1
		Zero Filling	On

[Result of Peak Picking]

No.	Position	Intensity	No.	Position	Intensity	No.	Position	Intensity	No.	Position	Intensity
1	3346.85	25.8278	2	3286.11	26.1388	3	3183.9	31.9825	4	3077.83	17.822
5	3017.09	31.5049	6	2990.09	33.2463	7	2960.2	32.2425	8	2933.2	30.0184
9	2874.38	35.7607	10	2687.32	40.853	11	2361.41	46.1118	12	1961.25	51.4987
13	1814.69	40.6359	14	1701.87	8.79292	15	1671.98	12.3455	16	1617.98	20.8418
17	1547.59	37.6702	18	1509.99	25.1798	19	1451.17	33.8374	20	1421.28	19.7925
21	1409.71	17.1302	22	1382.71	18.8702	23	1342.21	15.3393	24	1308.46	23.9639
25	1236.15	23.7005	26	1213.97	19.7392	27	1192.76	25.0113	28	1163.83	35.3991
29	1141.65	23.774	30	1100.19	33.5416	31	1071.26	22.8015	32	1044.26	23.1516
33	997.017	41.7009	34	944.949	35.6892	35	896.737	40.3044	36	872.631	42.3984
37	852.382	53.2422	38	808.992	59.2704	39	788.743	56.2663	40	727.996	33.7208
41	709.676	25.4879	42	698.105	22.9798	43	662.428	26.6234	44	644.108	33.369
45	593.968	37.927	46	531.293	48.6719	47	456.082	48.1124			

S3. FT-IR spectrum of [(H$_2$dap^{+})$_2$(tda^{2-})]·2H$_2$O, **2**.

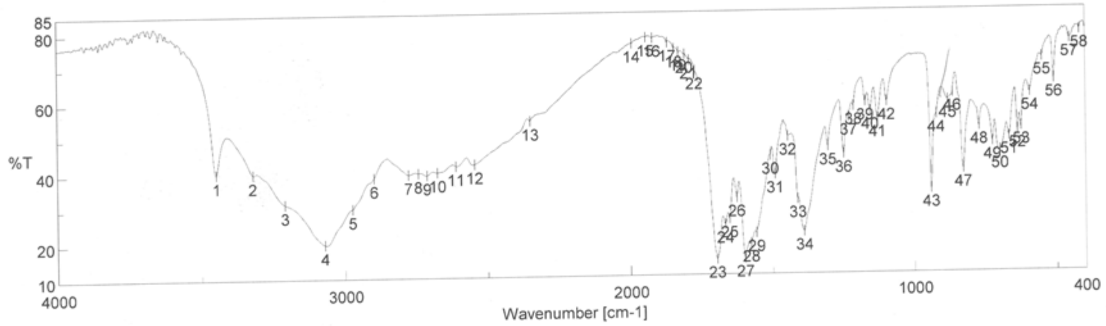

[Result of Peak Picking]

No.	Position	Intensity	No.	Position	Intensity	No.	Position	Intensity
1	3448.1	40.1847	2	3319.86	40.0203	3	3208.97	31.4661
4	3071.08	19.8961	5	2972.73	29.997	6	2896.56	38.6918
7	2776.03	39.5604	8	2742.28	39.9196	9	2711.42	39.3353
10	2674.78	40.1364	11	2610.18	41.8786	12	2545.58	42.2944
13	2348.87	54.3515	14	1992.11	76.0618	15	1942.93	77.7301
16	1920.75	77.612	17	1867.72	76.2356	18	1844.58	74.4644
19	1829.15	73.5038	20	1807.94	72.8565	21	1792.51	71.1755
22	1772.26	68.1629	23	1697.05	14.477	24	1667.16	24.3562
25	1652.7	25.9281	26	1626.66	31.8799	27	1597.73	14.7093
28	1576.52	18.9009	29	1558.2	21.842	30	1508.06	43.7791
31	1491.67	38.4566	32	1446.35	49.1221	33	1412.6	31.3347
34	1390.42	22.1055	35	1306.54	46.1441	36	1250.61	44.0036
37	1232.29	54.4746	38	1214.93	57.4845	39	1172.51	58.6732
40	1155.15	56.0337	41	1129.12	53.8631	42	1096.33	58.632
43	940.128	33.8832	44	921.807	54.961	45	882.274	58.944
46	864.917	60.9789	47	829.241	39.4057	48	772.351	51.4607
49	726.068	47.0549	50	700.034	44.4952	51	668.214	48.4501

S4. Thermogravimetric analysis (TGA) of [(H9Heade+)(Htda-)], **1**.

S4.1. Weight loss and data to estimate the final residual for **1**.

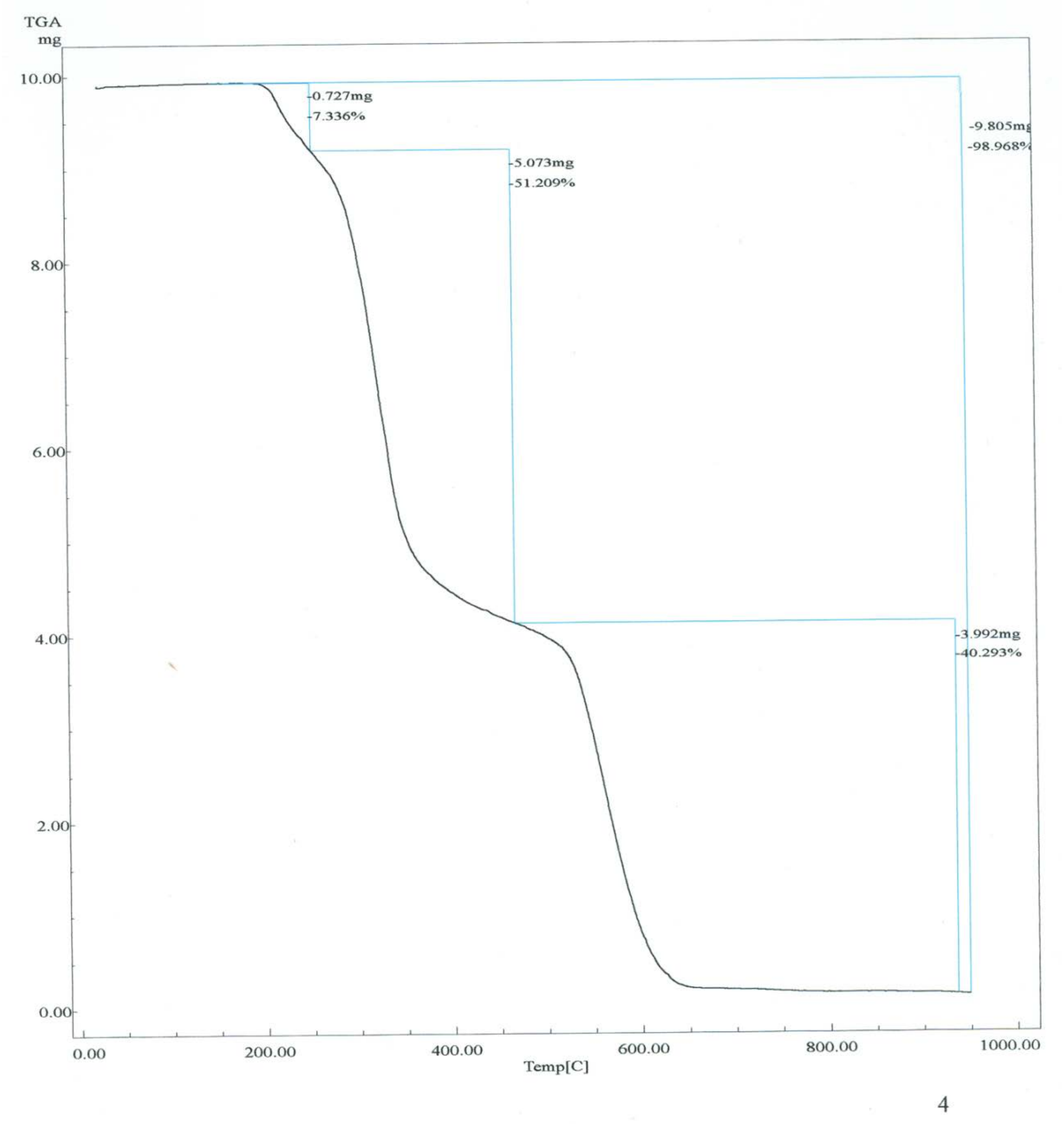

S4.2. FT-IR spectra of standard gases to identify evolved gases in TGA of **1**.

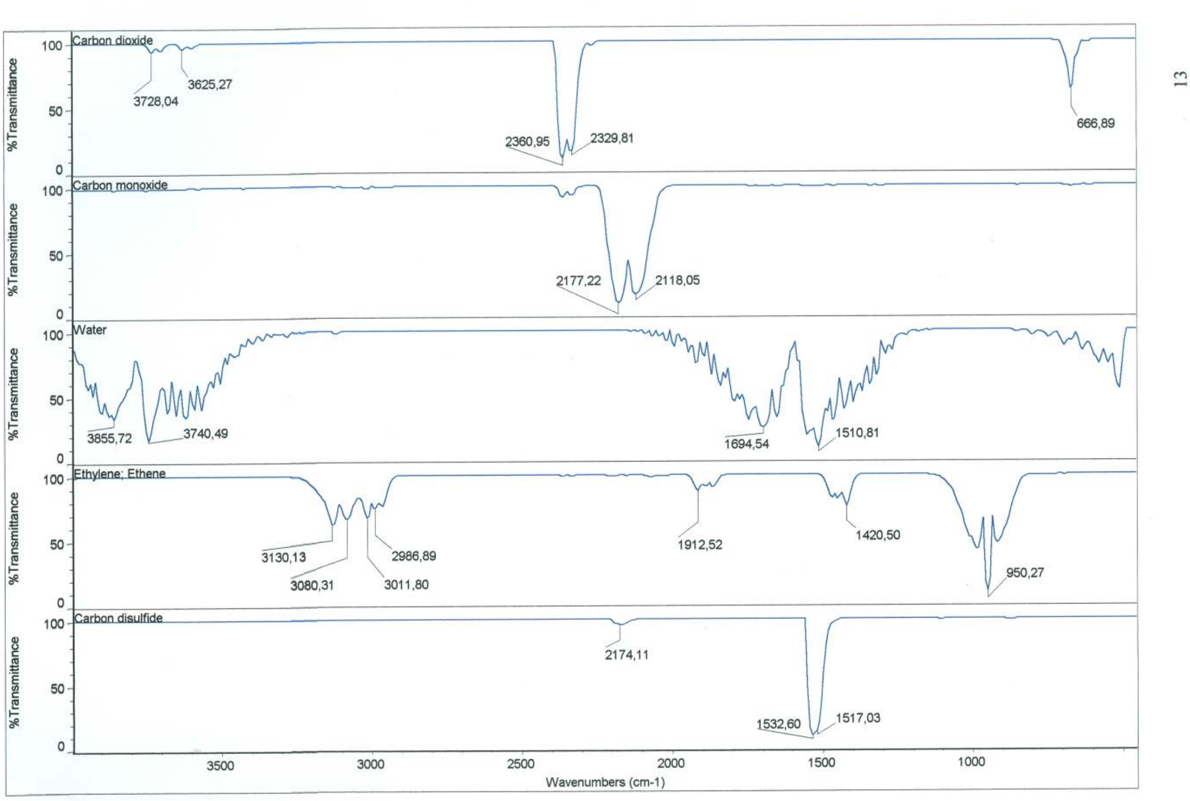

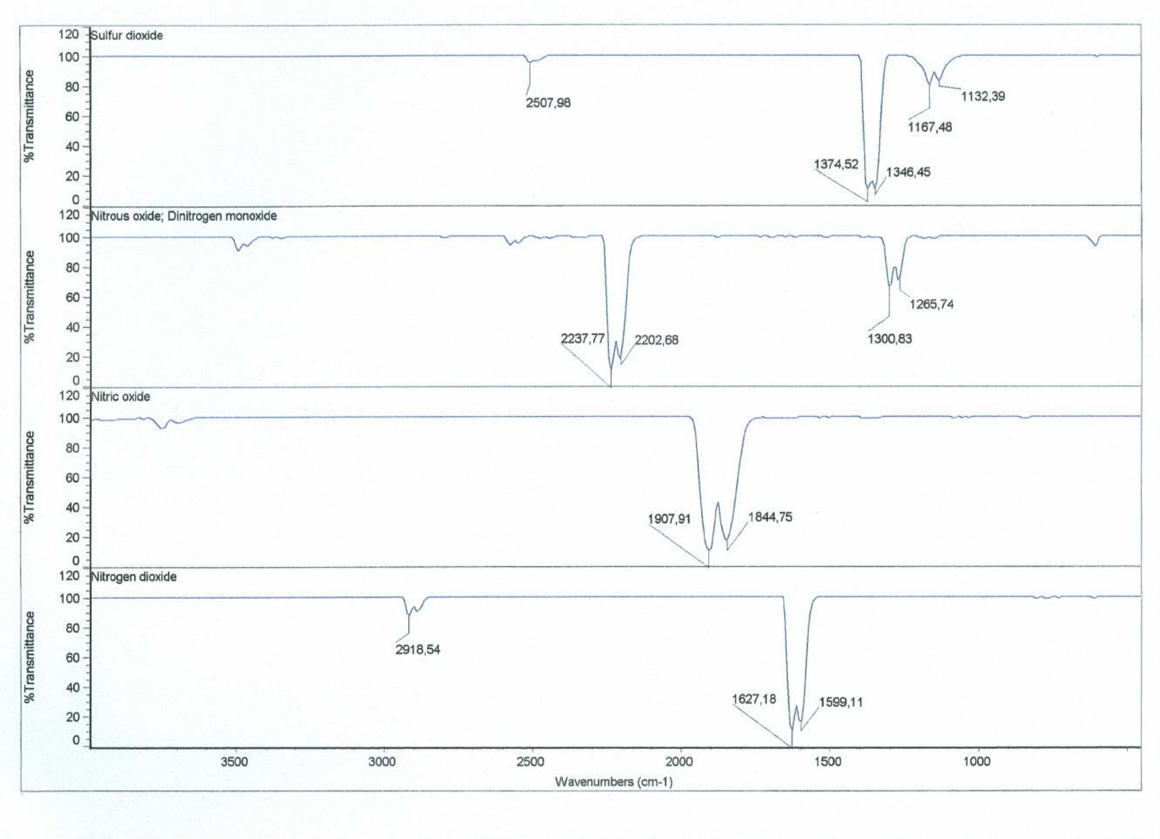

S4.3. Time-spaced FT-IR spectra to identify the evolved gases in the TGA of **1**.

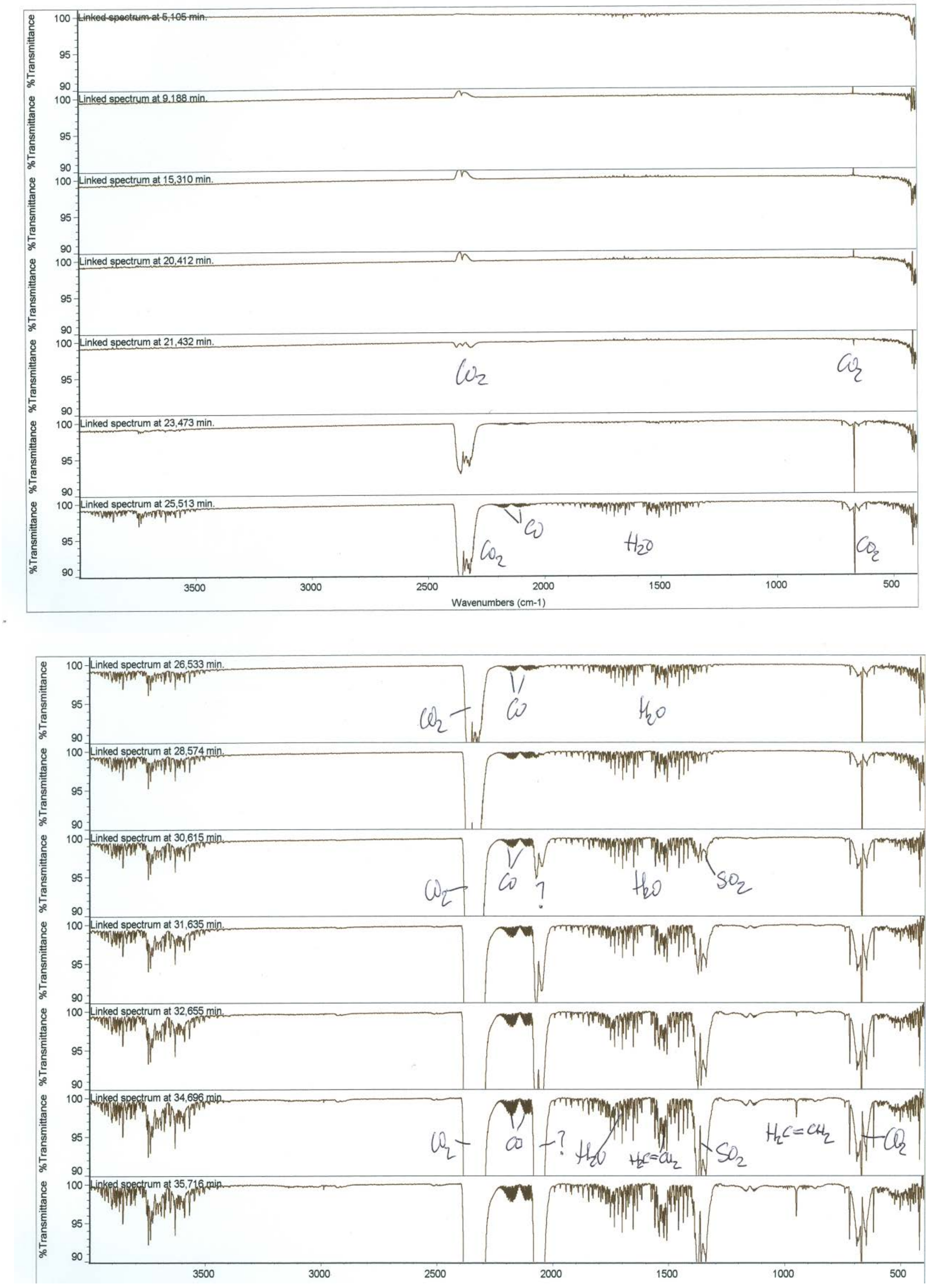

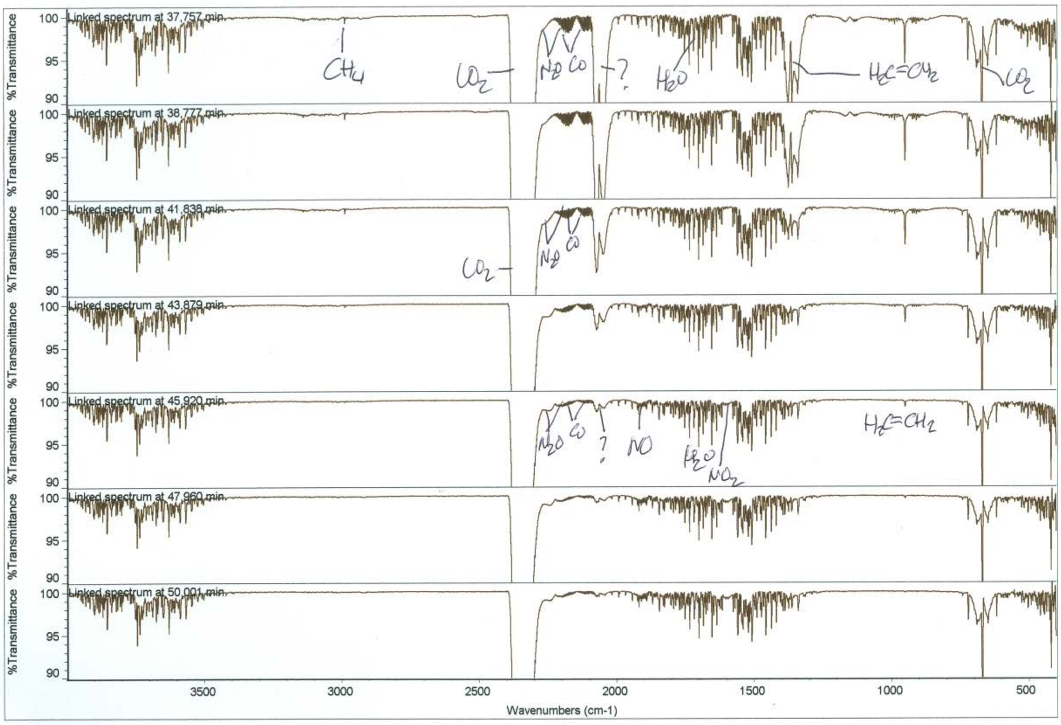
Linked spectrum at 37,757 min.
Linked spectrum at 38,777 min.
Linked spectrum at 41,838 min.
Linked spectrum at 43,879 min.
Linked spectrum at 45,920 min.
Linked spectrum at 47,960 min.
Linked spectrum at 50,001 min.
%Transmittance
100
95
90
3500
3000
2500
2000
1500
1000
500
Wavenumbers (cm-1)
CH4
CO2
N2O
CO
?
H2O
H2C=CH2
CO2
NO
NO2

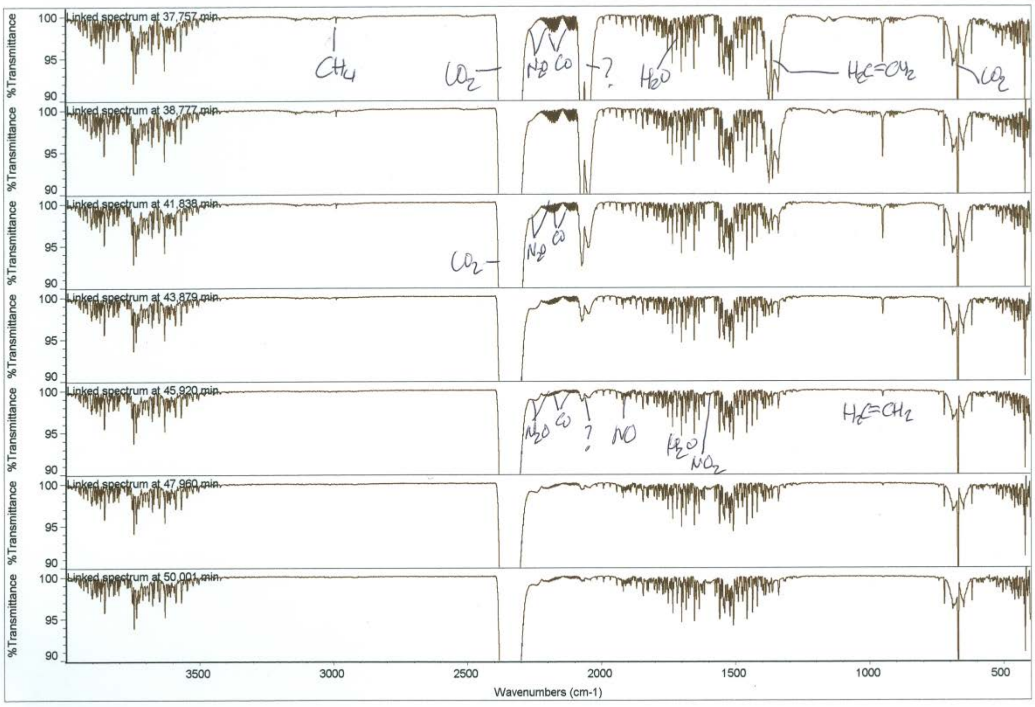
Linked spectrum at 37,757 min.
Linked spectrum at 38,777 min.
Linked spectrum at 41,838 min.
Linked spectrum at 43,879 min.
Linked spectrum at 45,920 min.
Linked spectrum at 47,960 min.
Linked spectrum at 50,001 min.
%Transmittance
100
95
90
3500
3000
2500
2000
1500
1000
500
Wavenumbers (cm-1)
CH4
CO2
N2O
CO
?
H2O
H2C=CH2
CO2
NO
NO2

9

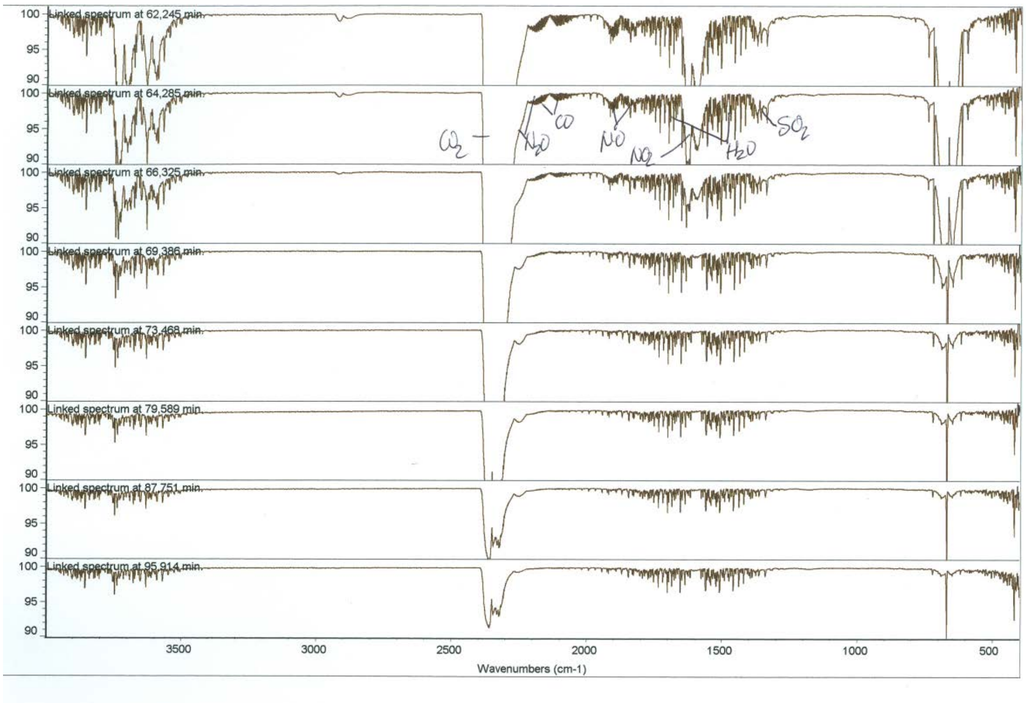

S4.4. Selected FT-IR spectra to identify the evolved gases in the TGA of 1. Second (up) and third (down) steps.

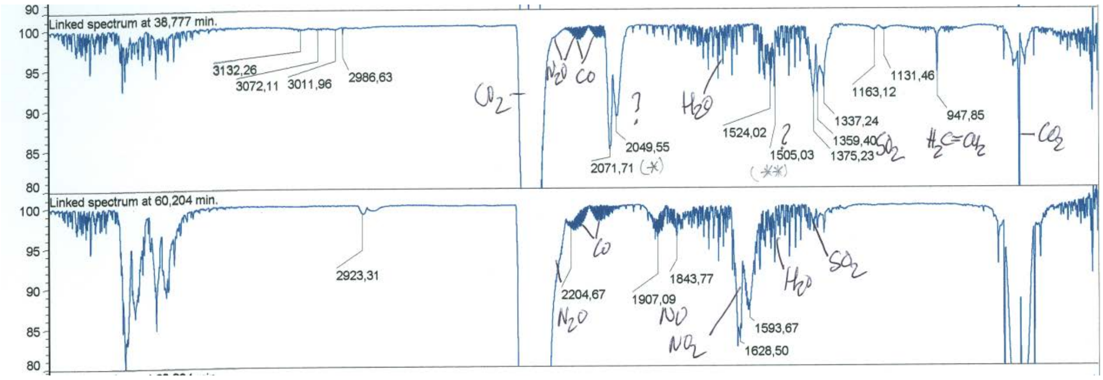

S4.5. Summary of results and Comments on TGA of **1.**

Step	Temperature °C	Time min	% Observed Weight Loss	Gases or final residue
1	**180-250**	14-23	7.336	H_2O, CO_2
2	250-**465**	23-44	51.209	H_2O, CO_2, CO, X**, SO_2, H_2C=CH_2, N_2O (t*)
3	465-650	44-66	40.293	H_2O, CO_2, CO, N_2O, NO, NO_2
Residue	650-**950**	93	1.032	Undetermined

* t = Trace amounts. ** X = Unidentified gas.

Coments:

Compound **1** exhibits rather high stability. Its decomposition initiates at 180°C. In the first step, it releases only H_2O and CO_2. The gases evolved during the second step primarily indicate the combustion of the Htda$^-$ counter-anion, potentially accompanied by a partial decomposition of the N9-(2-hydroxyerthyl) arm from the H9heade$^+$ cation, resulting in the production of ethylene and small quantities of N_2O. Subsequently, the final step, among others gases produced, the three common N-oxides, are generated, primarily due to the combustion of the purine moiety from 9heade. Notably absent are the typical peaks around 970 ad 930 cm^{-1}, associated with ammonia loss. Furthermore, this absence cannot be attributed solely to the high temperatures (>450 °C) of the third step, as all the three steps promote N-oxide formation. Indeed the thermogravimetric analysis of molecular 9heade (not shown here) revealed an initial weight loss >96% (215-420°C) releasing H_2O, CO_2, CO and N_2O (without produce ammonia), followed by a second step (420-625°C) producing these gases alongside NO and NO_2. The majority of the weight loss in compound **1** occurs below 700°C. Additionally, the chemical purity of the tested sample is affirmed by the low final residue (~1%) at 950 ˚C.

***Coments:**

Compound **1** initiates its decomposition at 180-250°C, resulting in a weight loss of 7.34% releasing H_2O and CO_2. This is followed by distinct phase between 250-465°C, accounting for 51.12 % of weight loss, characterized by the generation of H_2O, CO_2, CO, SO_2, H_2C=CH_2 and potentially HSCN along with some N_2O. A subsequent step from 465-650°C, responsible for 40.19% of weight loss, produces H_2O, CO_2, CO, and N-oxides (N_2O, NO and NO_2) excluding NH_3. A stable but minor residue forms (1.16% or 1.03% at 650 or 950°C, respectively). These findings are indicative of a partially overlapping combustion of both ligands and are consistent with the anticipated stability of the purine moiety of 9heade.

S5. Thermogravimetric analysis (TGA) $[(H_2dap^+)_2(tda^{2-})]\cdot 2H_2O$ (**2**).

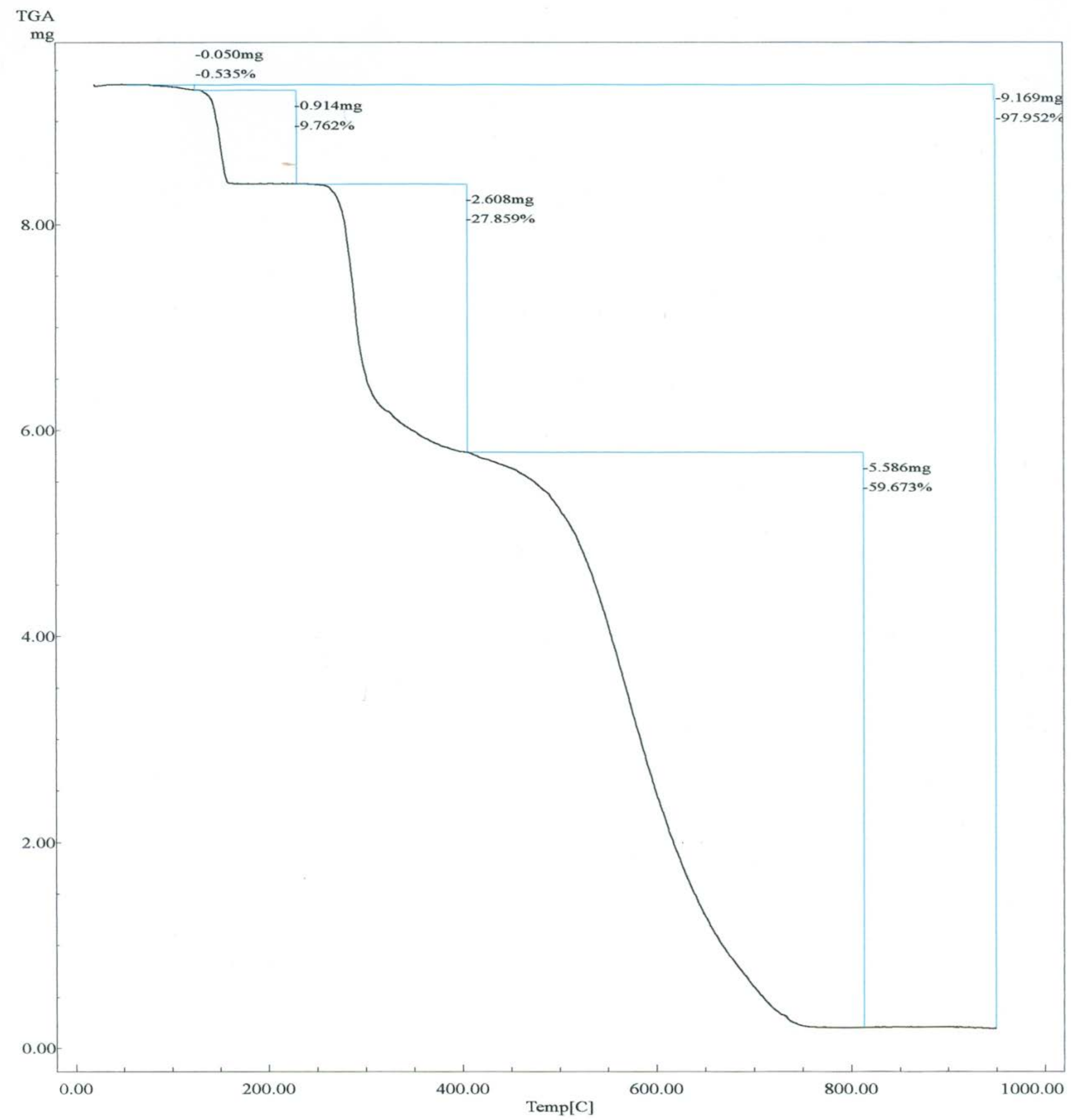

S5.2. FT-IR spectra of standard gases to identify evolved gases in TGA of **2**.

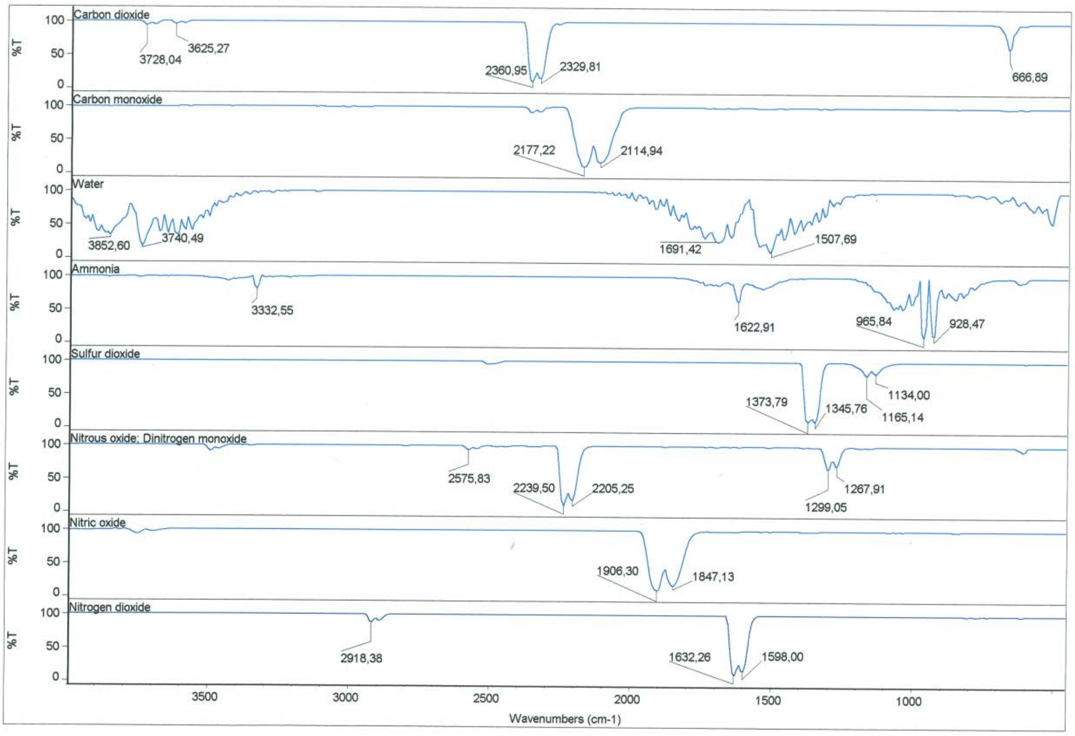

S5.3. Time-spaced FT-IR spectra to identify the evolved gases in the TGA of **1**.

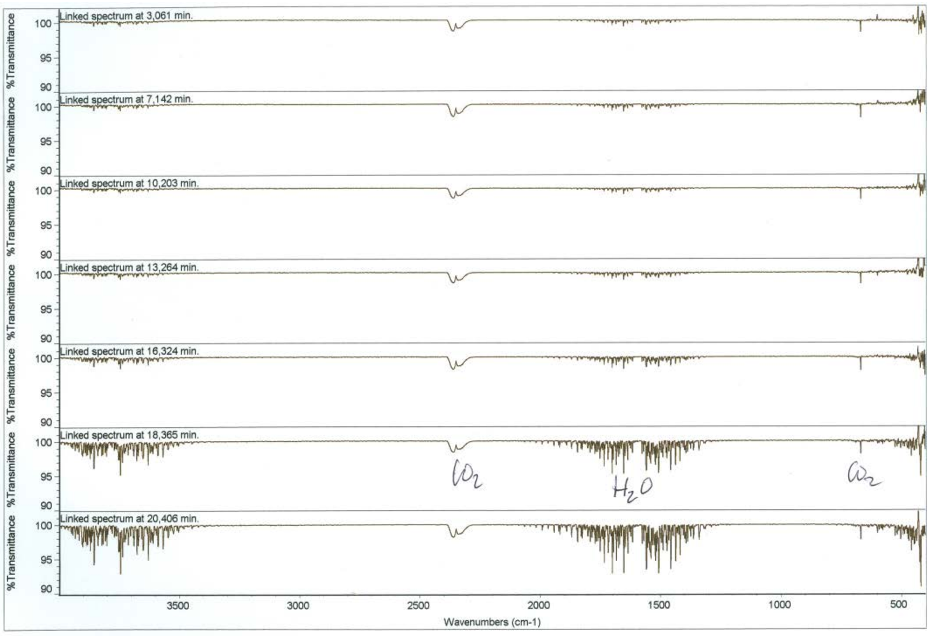

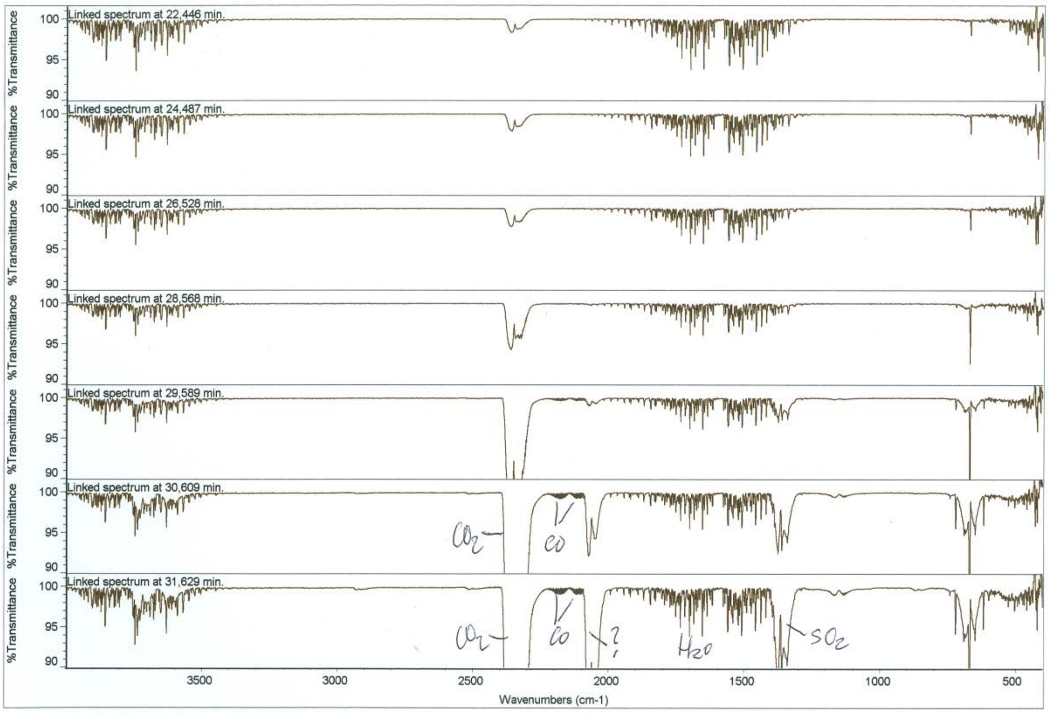
Linked spectrum at 22,446 min.
Linked spectrum at 24,487 min.
Linked spectrum at 26,528 min.
Linked spectrum at 28,568 min.
Linked spectrum at 29,589 min.
Linked spectrum at 30,609 min.
Linked spectrum at 31,629 min.
%Transmittance
Wavenumbers (cm-1)
CO_2
CO
?
H_2O
SO_2

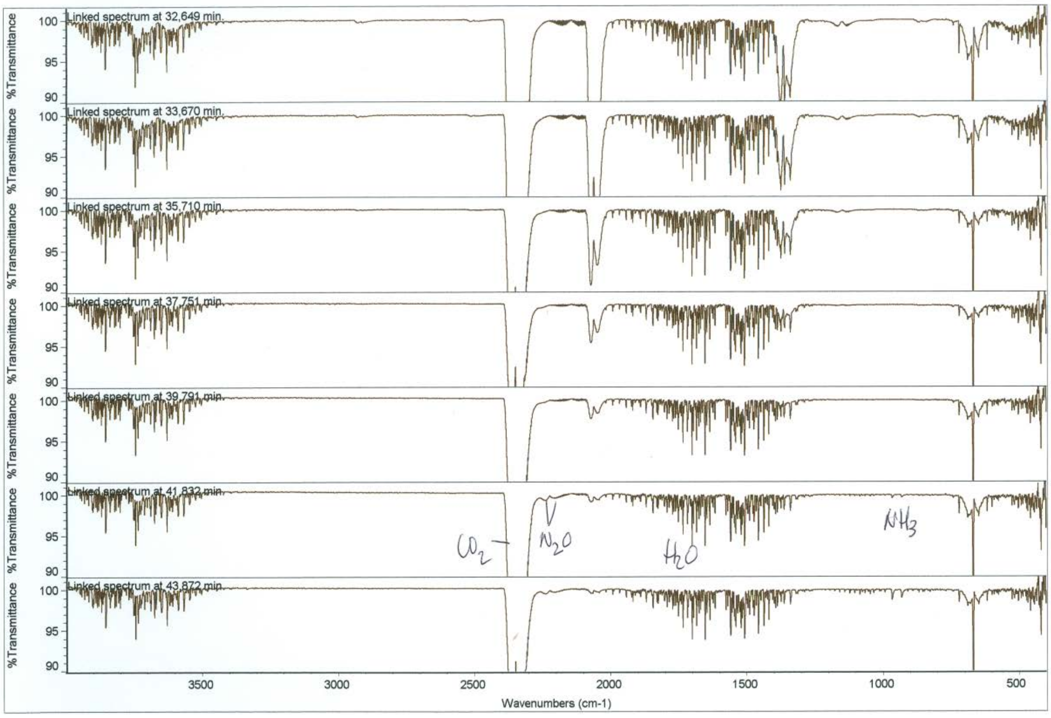
Linked spectrum at 32,649 min.
Linked spectrum at 33,670 min.
Linked spectrum at 35,710 min.
Linked spectrum at 37,751 min.
Linked spectrum at 39,791 min.
Linked spectrum at 41,832 min.
Linked spectrum at 43,872 min.
%Transmittance
Wavenumbers (cm-1)
CO_2
N_2O
H_2O
NH_3

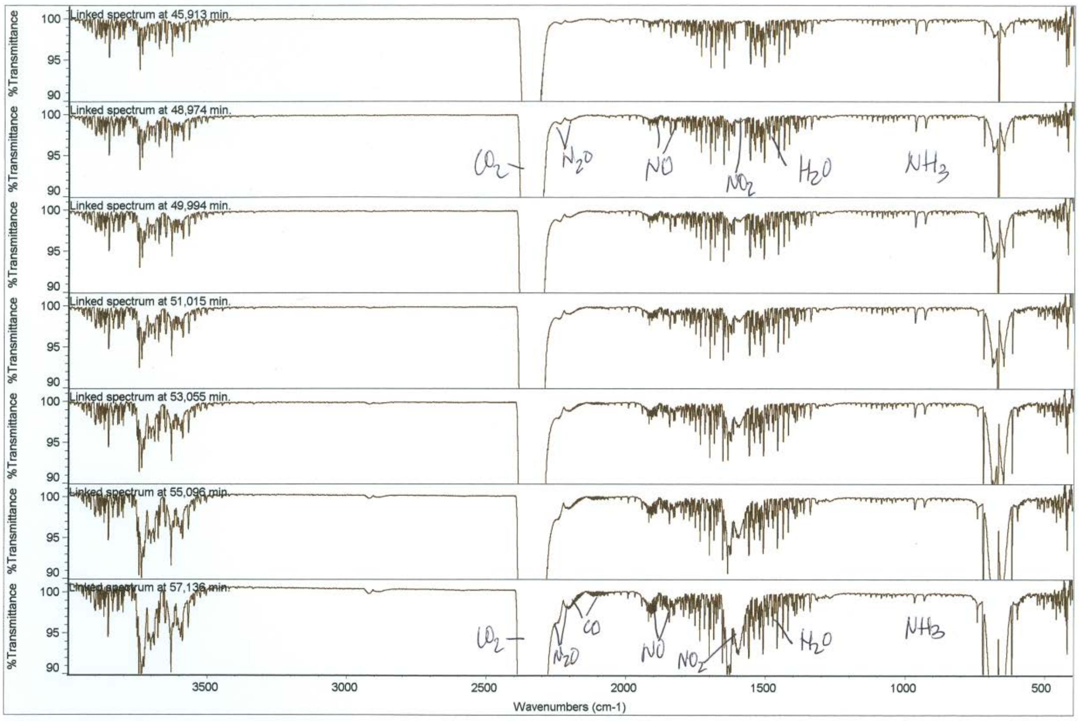
Linked spectrum at 45,913 min.
Linked spectrum at 48,974 min.
Linked spectrum at 49,994 min.
Linked spectrum at 51,015 min.
Linked spectrum at 53,055 min.
Linked spectrum at 55,096 min.
Linked spectrum at 57,136 min.
%Transmittance
Wavenumbers (cm-1)
CO_2
N_2O
NO
NO_2
H_2O
NH_3
CO

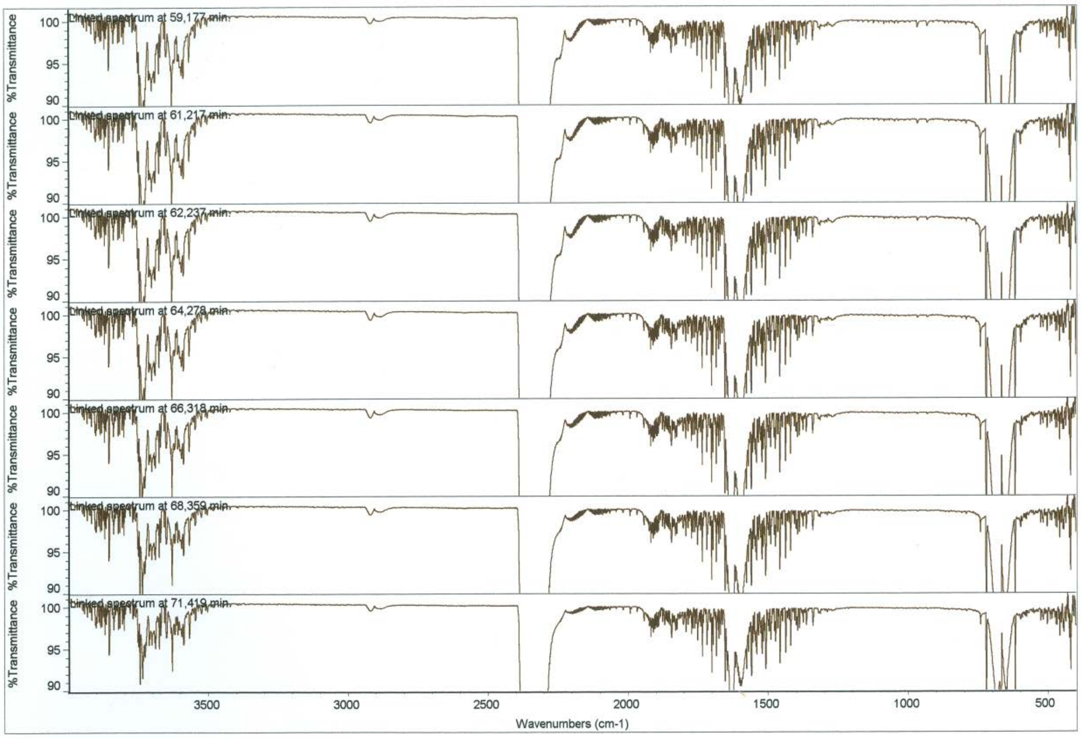
Linked spectrum at 59,177 min.
Linked spectrum at 61,217 min.
Linked spectrum at 62,237 min.
Linked spectrum at 64,278 min.
Linked spectrum at 66,318 min.
Linked spectrum at 68,359 min.
Linked spectrum at 71,419 min.
%Transmittance
Wavenumbers (cm-1)

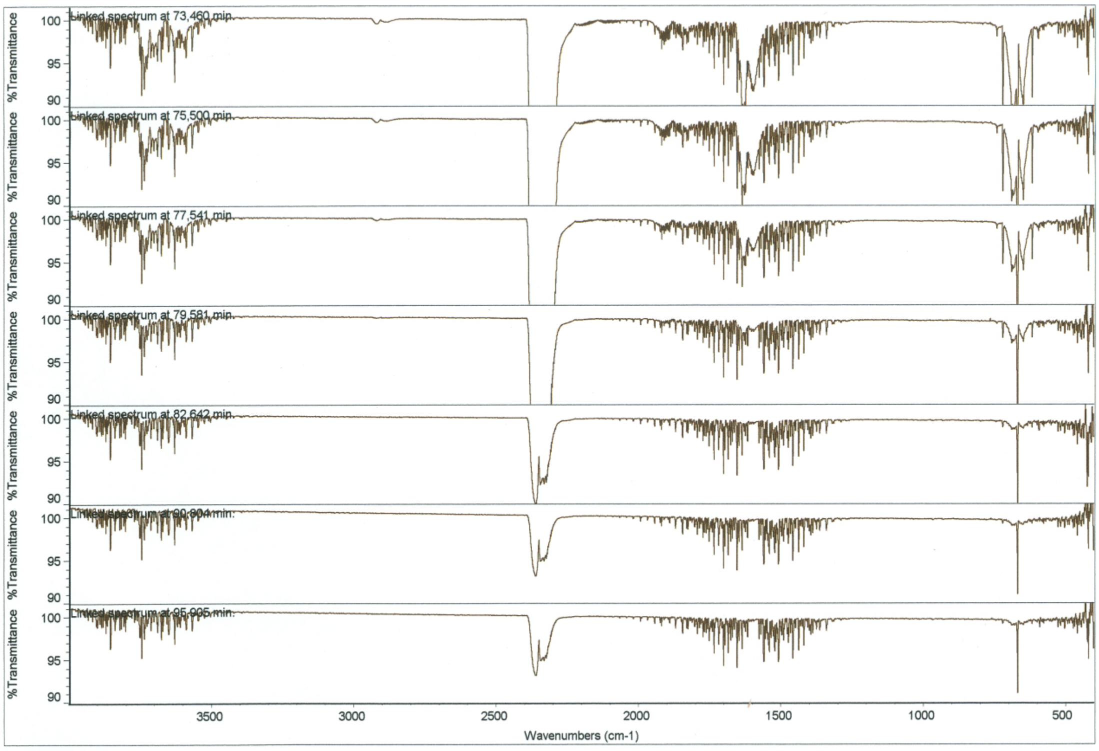

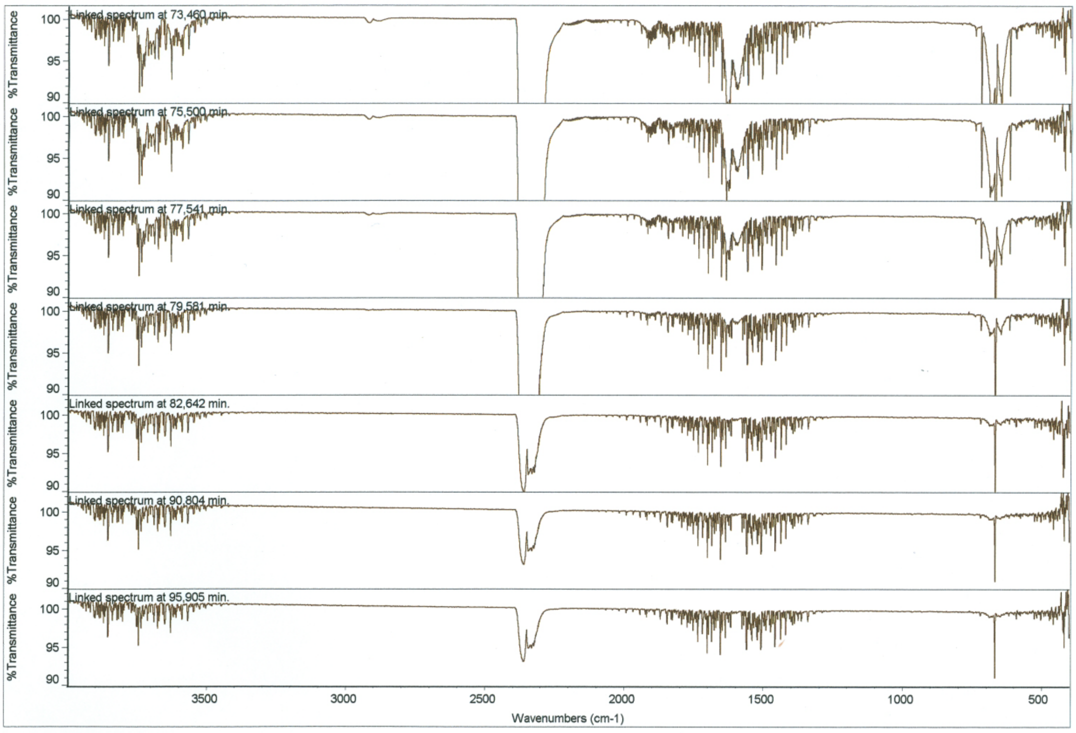

S5.4. Selected FT-IR spectra to identify the evolved gases in the TGA of **2**. Second (up) and third (down) steps.

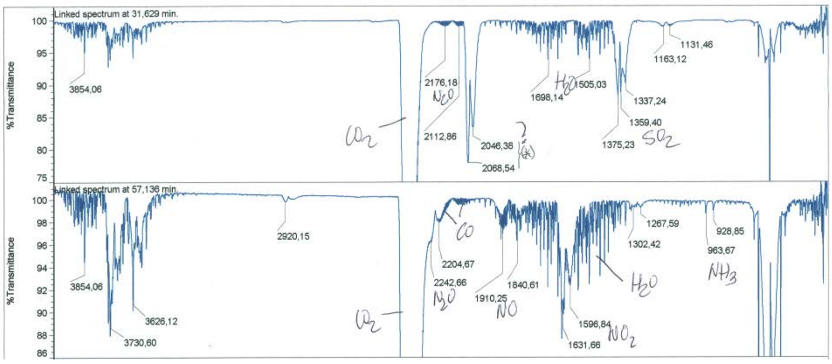

S**5.5** Summary of results and Comments on TGA of **2.**

Step	Temperature ºC	Time min	Lost weight (%)		Gases or final residue
			Observed	Calculated	
0	r.t.-**135**	0-12	0.535	*Humidity*	H_2O, CO_2 (t*)
1	135-**180**	12-18	9.762	7.047	2 H_2O, CO_2 (t*)
2	180-**400**	18-39	27.859	30.452	H_2O, CO_2, CO, X**, SO_2
3	400~800	39-82	59.673	62.142	H_2O, CO_2, CO,<br>NH_3, N_2O, NO, NO_2
Residue	950	93	2.048	0	Undetermined

*t = Trace amounts. ** X = Unidentified gas.

Comments:

At the outset, the analyzed sample of compound **2** (9.361 mg) registers a minimal weight loss (referred to as step 0, possibly attributable to moisture < 0.6%). The thermal breakdown of compound **2** primarily commences by eliminating its tightly bound water content (termed step 1, 135-180ºC, calculated for two water molecules only). Subsequently, the organic combustion takes place across two new steps. The first stage (step 2, 180-400ºC) shows a weight loss closely aligning with the anticipated value for the combustion of the tda^{2-} ion (30.45%), with the evolved gasses reasonably corroborating this process. Notably, an unidentified gas 'X' is released during this stage, a phenomenon also observed in the thermogravimetric analysis of free H_2tda acid (not presented here). The weight loss in the final step 3 (400-800ºC) similarly matches the estimated value for two H_2dap^+ ions (62.142%). Once more, the evolved gasses align with these observations. Collectively, these steps result in a residual mass of 2% (at 900°C). The cumulative weight loss from steps 1-3, alongside the residual mass (at 950ºC) nearly corresponds to the total sample weight projected for compound **2** (excluding moisture), accounting for approximately 99.342%.

S.6. Thermogravimetric analyses (TGA) of used reagents in this wok.

S6.1.TGA of 2,2’-Thiodiacetic acid, H_2tda (powder sample, 98%).

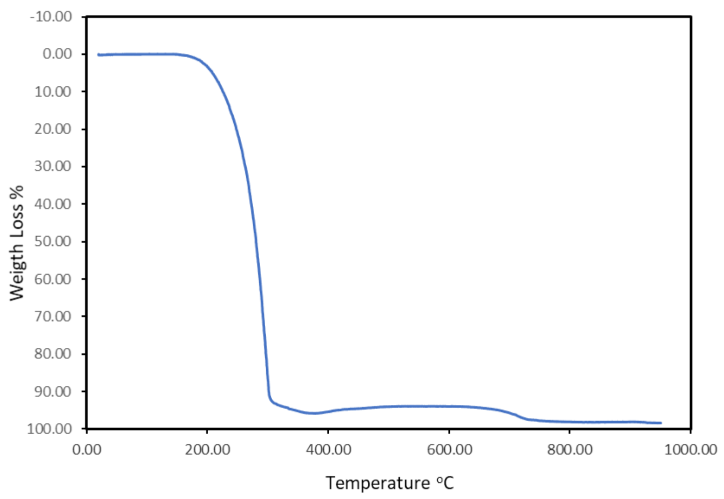

Step/Residue	Temperature °C	Time min	Weight* (%)	Gases or Residue (R)
1	130-325	12-31	–93.886	H_2O, CO_2, CO, SO_2, CS_2, X**
2	325-380	31-36	–1.851	H_2O, CO_2, CO, H_2CO, SO_2, CS_2, X**
R-380	380	36	–4.263	
3	380-550	36-54	+ 1.855	H_2O, CO_2, CO, H_2CO, SO_2, CS_2, X**
4	550-830	54-83	–1.222	H_2O, CO_2, CO, H_2CO, SO_2
R-830	830	83	–1.89	Unidentified
R-950	950	93	–1.64	Unidentified

*Lost (–) or gained (+) weight. ** Unidentified gas.

Comments:

The TGA analysis of this compound reveals an initial step with a weight loss of approximately 94%, where the formation of CS_2 and SO_2 was identified as sulfur-gases. The observed saw-tooth pattern in the first derivative of the weight loss plot versus temperature aligns with the reported melting point for this acid (128-131ºC). In the subsequent step, the presence of formalin was also noted. The remaining material (~ 4.2%) shows an increase in weight before undergoing further decomposition resulting in a final residue of less than 2%, which aligns well with the claimed purity stated by the supplier.

Selected reference: Vinciguerra, V., Bucci, R., Marini, F., Napoli, A., Thermal behaviour of iminodiacetic, oxydiacetic and thiodiacetic acids. *J. Therm. Anal. Calorim.* **2006**, *83*, 475–478. https://doi.org/10.1007/s10973-005-6939-6

S6.2. TGA of 2,6-diaminopurine, Hdap (powder sample, 98%).

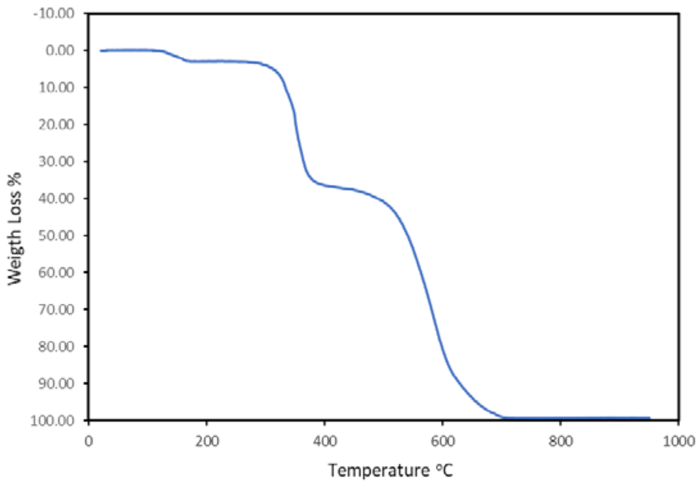

Step/Residue	Temperature °C	Time min	Weight* (%)	Gases or Residue (R)
1	115-220	9-16	– 3.119	H_2O, CO_2(t),
2	240-335	16-33	– 9.097	H_2O, CO_2
3	335-435	36-54	– 25.226	H_2O, CO_2, CO(t), **NH_3**, N_2O
4	435-775	54-83	– 61.288	H_2O, CO_2, CO(t), NH_3, N_2O, NO, NO_2
R-830	775	83	0.712	Unidentified

*Lost (–) or gained (+) weight.

Comments:

The initial step primarily exhibits water loss (along with some CO_2). The second step begins above 240°C, without notable production of N-gases. The weight loss becomes substantial in this phase about 300 °C, in line with its reported melting point (302°C). Subsequent steps generate water, CO_2, some CO, NH_3 and N-oxides. Our findings also demonstrate that steps 3 and 4 (> 85% of weight loss) notably generate N-gases (including ammonia) and result in < 1% residue. As a result, we infer that the sample under study contains approximately 3% water.

S6.3. TGA of N9-(2-hydroxyethyl)adenine, 9heade (powder sample, 98%).

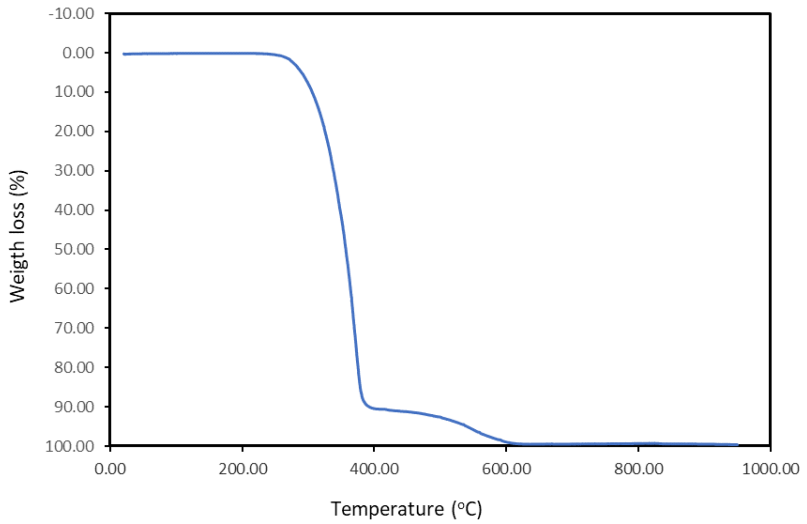

Step/Residue	Temperature °C	Time min	Weight* (%)	Gases or Residue (R)
1	215-420	18-39	– 96.431	H_2O, CO_2, CO, N_2O (t)
2	420-625	39-63	– 3.380	H_2O, CO_2, CO(t), N_2O, NO,NO_2
R-625	625	63	0.176	Unidentified
3	625-950	63-95	0.679	H_2O, CO_2, CO(t), N_2O, NO,NO_2, SO_2 (i)
R-950	950	95	0.855	Unidentified

*Lost (–) or gained (+) weight. (t) = Small quantity. (i) Unexpected gas, attributed to impunity.

Comments:

The vast majority of the sample (>96%) undergoes melting and decomposition in the initial step, aligning well with the reported melting point for 9heade (243°C). Once again, the saw-tooth pattern in the first derivative of the weight loss plot versus temperature concurs with the reported melting point for this synthetic purine nucleoside (242 or 240-244°C). The resulting material undergoes nearly complete decomposition resulting in a residue (<0.2%) at 625°C. This residual material further gains weight, culminating in a final residue of <1% at 950ºC. Additionally, during this last step, the production of SO_2 from an impurity or contaminant is observed. Nevertheless, our findings align with the supplier's guaranteed purity (98%). Interestingly, no ammonia is detected among the evolved gases, in contrast to the typical presence observed in the TGA of adenine or 2,6-diaminopurine (see **S6.2**) and near all of its metal complexes.

S7. DSC curve for [(H9Heade^{+})(Htda^{-})], **1**.

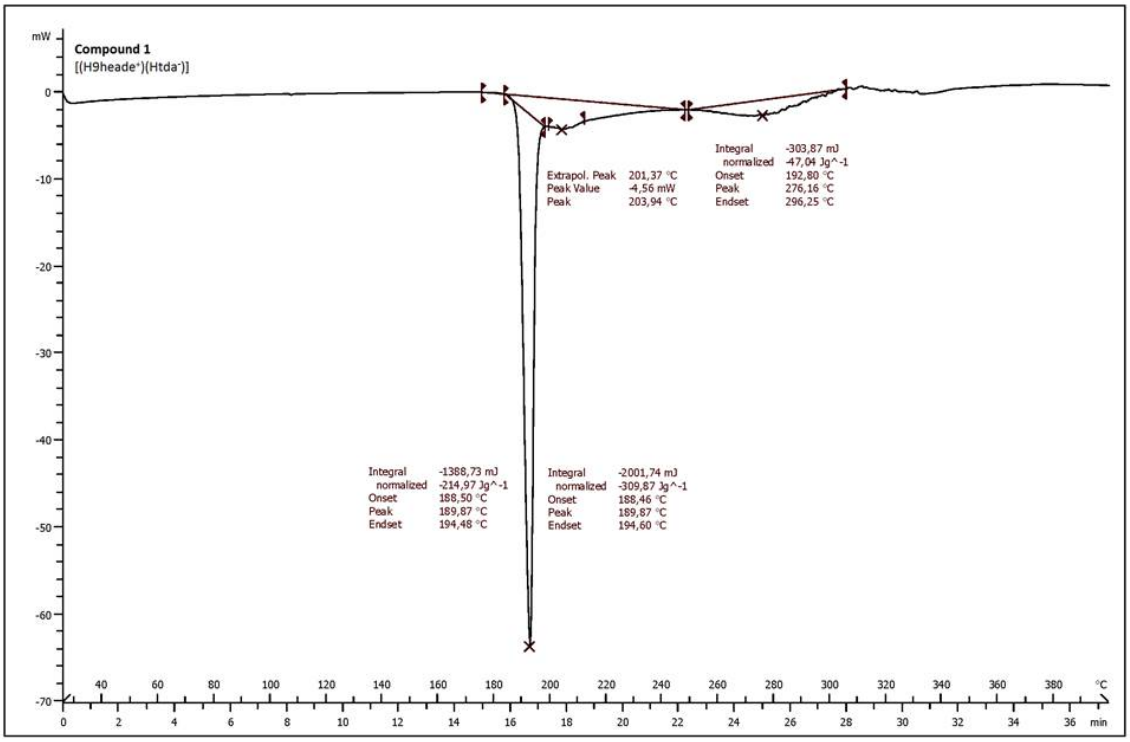

S8. DSC curve for $[(H_2dap^+)_2(tda^{2-})]\cdot 2H_2O$, **2**.

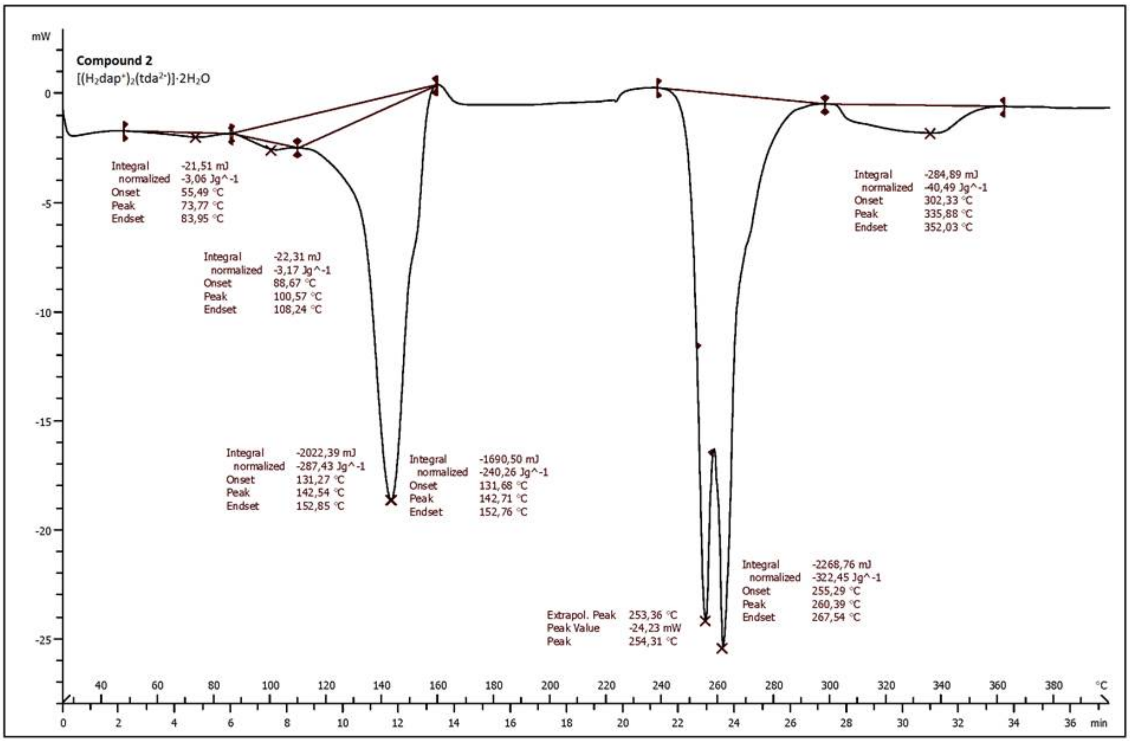

S9. ^{1}H NMR spectrum in D_2O of **1**.

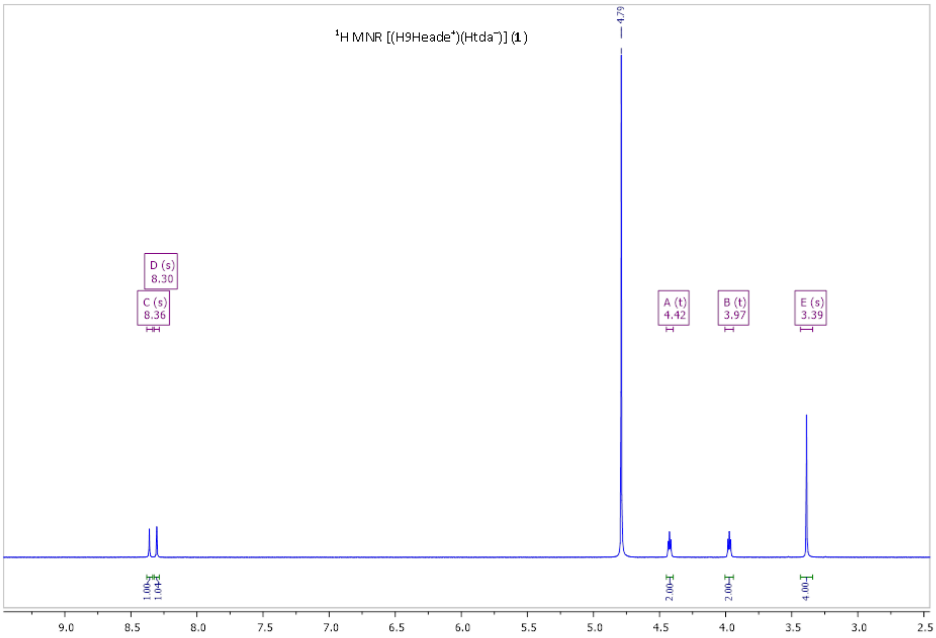

S10. ^{1}H NMR spectrum in D_2O of **2**.

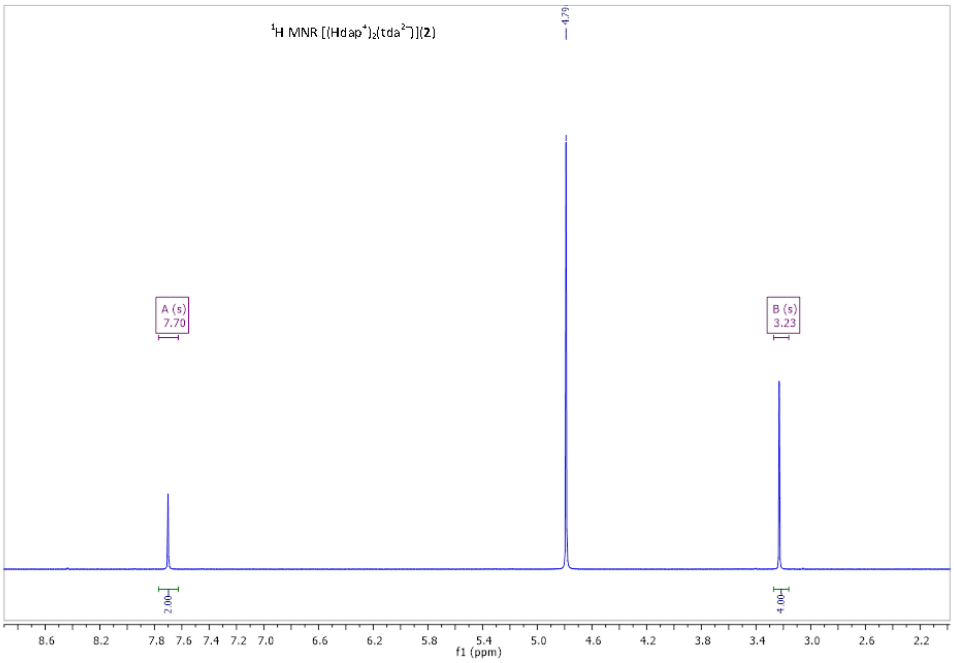

S11. ^{13}C NMR spectrum in D_2O of **1**.

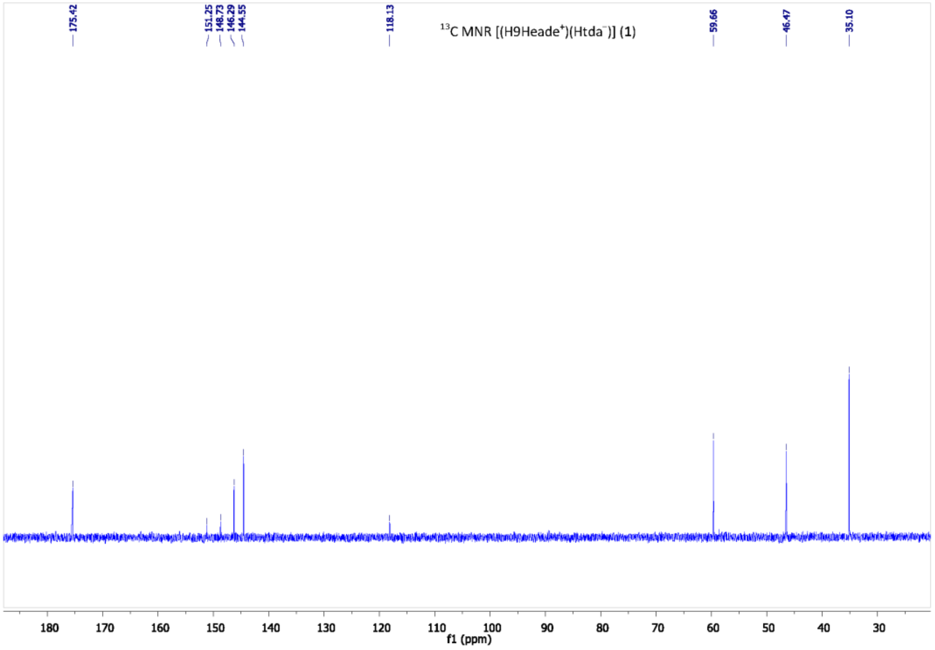

S12. ^{13}C NMR spectrum in D_2O of **2**.

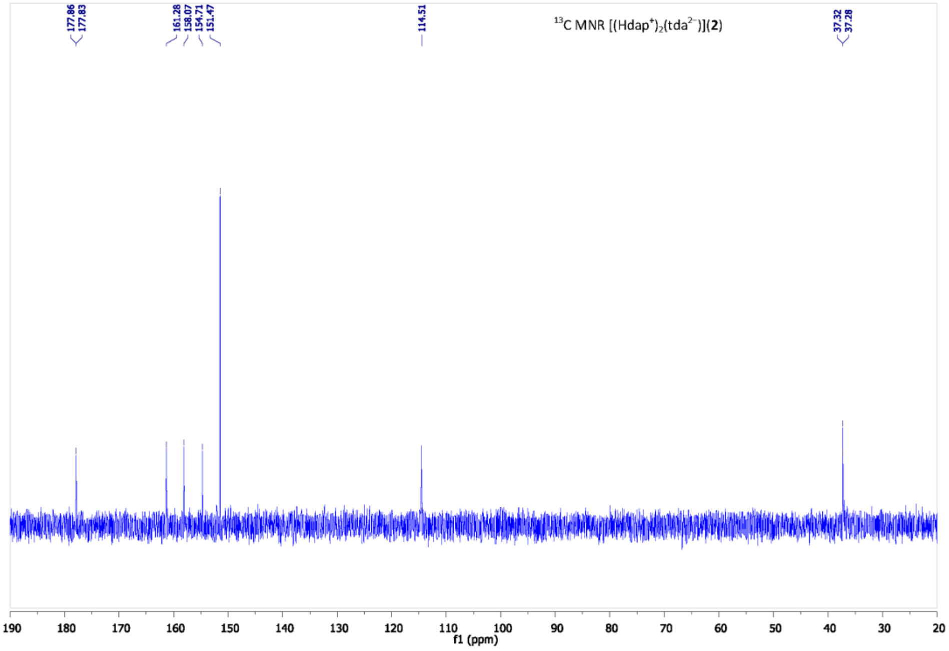

Supplement: Supplementary file 1 [file ijms-24-17381-s001.zip › ijms-2745950-supplementary.pdf]
